# Increased abscisic acid levels in transgenic maize overexpressing *AtLOS5* mediated root ion fluxes and leaf water status under salt stress

**DOI:** 10.1093/jxb/erv528

**Published:** 2016-01-07

**Authors:** Juan Zhang, Haiyue Yu, Yushi Zhang, Yubing Wang, Maoying Li, Jiachang Zhang, Liusheng Duan, Mingcai Zhang, Zhaohu Li

**Affiliations:** State Key Laboratory of Plant Physiology and Biochemistry, College of Agronomy and Biotechnology, China Agricultural University, Beijing 100193, China

**Keywords:** ABA, *AtLOS5*, ion fluxes, maize, salt stress, water uptake.

## Abstract

Abscisic acid (ABA) is a vital cellular signal in plants, and effective ABA signalling is pivotal for stress tolerance. *AtLOS5* encoding molybdenum cofactor sulphurase is a key regulator of ABA biosynthesis. Here, transgenic *AtLOS5* plants were generated to explore the role of *AtLOS5* in salt tolerance in maize. *AtLOS5* overexpression significantly up-regulated the expression of *ZmVp14-2*, *ZmAO*, and *ZmMOCO*, and increased aldehyde oxidase activities, which enhanced ABA accumulation in transgenic plants under salt stress. Concurrently, *AtLOS5* overexpression induced the expression of *ZmNHX1*, *ZmCBL4*, and *ZmCIPK16*, and enhanced the root net Na^+^ efflux and H^+^ influx, but decreased net K^+^ efflux, which maintained a high cytosolic K^+^/Na^+^ ratio in transgenic plants under salt stress. However, amiloride or sodium orthovanadate could significantly elevate K^+^ effluxes and decrease Na^+^ efflux and H^+^ influx in salt-treated transgenic roots, but the K^+^ effluxes were inhibited by TEA, suggesting that ion fluxes regulated by *AtLOS5* overexpression were possibly due to activation of Na^+^/H^+^ antiport and K^+^ channels across the plasma membrane. Moreover, *AtLOS5* overexpression could up-regulate the transcripts of *ZmPIP1:1*, *ZmPIP1:5*, and *ZmPIP2:4*, and enhance root hydraulic conductivity. Thus transgenic plants had higher leaf water potential and turgor, which was correlated with greater biomass accumulation under salt stress. Thus *AtLOS5* overexpression induced the expression of ABA biosynthetic genes to promote ABA accumulation, which activated ion transporter and PIP aquaporin gene expression to regulate root ion fluxes and water uptake, thus maintaining high cytosolic K^+^ and Na^+^ homeostasis and better water status in maize exposed to salt stress.

## Highlight

*AtLOS5* overexpression modulated ABA biosynthesis together with ion transporter and PIP aquaporin expression to mediate ion fluxes and water status of maize under salt stress.

## Introduction

Salt stress is a major abiotic stress of crop plants worldwide, with intensive irrigation leading to salinity in semi-arid and arid regions, and about one-third of the world’s irrigated land being severely affected by secondary salinization (Munns and Tester, 2008). Unfortunately, most staple crops are relatively salt sensitive, so salinity is becoming a great threat to sustainable food production (Flowers, 2004). To cope with salt stress, plants have evolved complex and integrated responses including biochemical and physiological processes and morphological and developmental changes (Flowers, 2004; Munns and Tester, 2008). Among the evolutionarily conserved pathways, abscisic acid (ABA) signalling has been identified as a central regulator of abiotic stress response in plants, triggering major changes in gene expression and adaptive physiological responses (Zhang *et al.*, 2006). Salinity enhances ABA accumulation in vegetative tissues, and exogenous ABA may alleviate the deleterious effects of salt stress in several plant species (Chen *et al.*, 2006; Park *et al.*, 2008). Thus manipulating salt-induced ABA accumulation has great agronomic potential for improving salt tolerance in crops.

The key ABA biosynthesis enzymes such as ZEP, NCED3, ABA3, and AAO3 have been identified, and expression of their corresponding genes is induced either by abiotic stress or by ABA (Xiong *et al.*, 2001; Park *et al.*, 2008). Moreover, genetic engineering using these genes has improved plant salt tolerance. For example, overexpression of the 9-*cis*-epoxycaroteniod dioxygenase gene (*NCED*) increased ABA concentration and salt tolerance in tobacco (Zhang *et al.*, 2009) and creeping bentgrass (Aswath *et al.*, 2005). Transgenic Arabidopsis plants overexpressing the zeaxanthin epoxidase gene show higher ABA concentrations and greater tolerance of salt stress (Park *et al.*, 2008).

The oxidation of abscisic aldehyde to ABA is catalysed by aldehyde oxidase (AO) in the last step of the ABA biosynthesis pathway. Sulphuration of the molybdenum cofactor (MoCo) is an essential step for activating AO, and MoCo sulphurase (MCSU or LOS5) transfers the sulphur ligand to abscisic aldehyde-bound MoCo, which is involved in regulating ABA biosynthesis (Xiong *et al.*, 2001). Thus, the expression of *OsMCSU* and *AtLOS5* is induced by drought, salt, and ABA. *AtLOS5* overexpression can increase ABA accumulation and improve drought tolerance in transgenic maize (Lu *et al.*, 2013), soybean (Li *et al.*, 2013), and cotton (Yue *et al.*, 2012). Although *AtLOS5* overexpression can improve salt tolerance via increasing ABA accumulation and antioxidant systems in sweet potato (Gao *et al.*, 2011), little is known about the roles of overexpressing ABA biosynthesis genes in staple crops such as maize under salt stress.

Maize is considered to be a moderately salt-sensitive crop, although its salt tolerance exhibits intraspecies variability (Pitann *et al.*, 2013). Maize is grown on >30 Mha annually in China, especially in China’s semi-arid and arid regions where water shortage means there is a need for irrigation. Nevertheless, intensive irrigation results in salinity, which affects plant morphological attributes and disturbs plant metabolic activities that lower maize grain yields (Zhang *et al.*, 2006). Recently, physiological and genetic mechanisms regulating salt tolerance have been established, thereby identifying suitable candidate genes for engineering more salt-tolerant crops (Munns and Tester, 2008). However, there are few reports on the modulation of stress-induced genes in maize to allow adaptation to salt stress.

The detrimental effects of salinity on plant growth and productivity are mainly caused by osmotic stress, ion toxicity, and oxidative stress (Munns, 2002; Munns and Tester, 2008). To cope with the problem, plants must readjust their water or osmotic homeostasis, ion balance, and antioxidant systems (Horie *et al.*, 2011). Decreased root hydraulic conductivity (*L*pr) is one of the first plant responses to salt stress (Boursiac *et al.*, 2005; Horie *et al.*, 2011), which is achieved by a down-regulation of the mRNA and protein level of most aquaporins (Qian *et al.*, 2015). In contrast to down-regulation of aquaporins, salt stress increases the expression of plasma membrane intrinsic protein (PIP) aquaporins, while *L*pr decreases in maize roots (Marulanda *et al.*, 2010). *ZmPIP* genes are transiently induced by 100mM NaCl (Zhu *et al.* 2005), but 150mM or 200mM NaCl represses the *ZmPIP* and *ZmTIP* genes in maize roots (Wang *et al.*, 2003). Exogenous ABA enhances *L*pr in maize under water or salt stresses, and ABA can also increase the expression of *PIP* genes (Ruiz-Lozano *et al.*, 2009; Marulanda *et al.*, 2010). Therefore, salt stress dramatically influences water uptake in roots, and regulation of water channels is important in plant responses to salt stress.

NaCl toxicity is often attributed to Na^+^ concentrations, and Na^+^ toxicity is associated with the ability to maintain the acquisition and distribution of K^+^ in plants (Shabala and Cuin, 2008; Anschütz *et al.*, 2014). To achieve salt tolerance, maintaining intracellular K^+^ and Na^+^ homeostasis is crucial for cytosolic enzyme activity and membrane potential and stability, and this process involves salt stress-responsive signal transduction, expression of the corresponding genes, and accumulation of compatible solutes (Anschütz *et al.*, 2014). Many studies have shown that ABA improves the activity and/or the expression of tonoplast Na^+^/H^+^ antiporter, which achieves K^+^/Na^+^ homeostasis (Shi and Zhu, 2002; Fukuda *et al.*, 2011).

According to the model for NaCl stress induction of ABA biosynthesis described by Barrero *et al.* (2006), increased expression of the indicated genes in ABA biosynthesis can improve the ABA level. The *AtLOS5* gene encodes MoCo sulphurase involved in regulation of *AAO3* expression in the ABA biosynthesis pathway (Xiong *et al.*, 2001), and *AtLOS5* overexpression increases ABA concentrations and enhances drought tolerance in several crops (Yue *et al.*, 2012; Li *et al.*, 2013; Lu *et al.*, 2013). However, little information is available on the modulation of ABA biosynthesis to regulate water uptake and ion homeostasis in maize under salt stress. This study used transgenic *AtLOS5* plants to explore the roles of *AtLOS5* in regulating the expression profiles of ABA biosynthetic and responsive genes under NaCl stress. Moreover, a non-invasive ion flux technique was used to analyse the NaCl-induced alternations of ion fluxes between transgenic and wild-type (WT) plants. Furthermore, we examined the effects of salt shock and plasma membrane (PM) transport inhibitors on Na^+^ and H^+^ fluxes in roots of transgenic and WT plants. Combined with the analysis of root hydraulic properties and PIP aquaporin gene expression, the aim was to clarify the role of *AtLOS5* overexpression in maize in salt stress responses.

## Materials and methods

### Plant growth and treatments

**Fig. 1.**
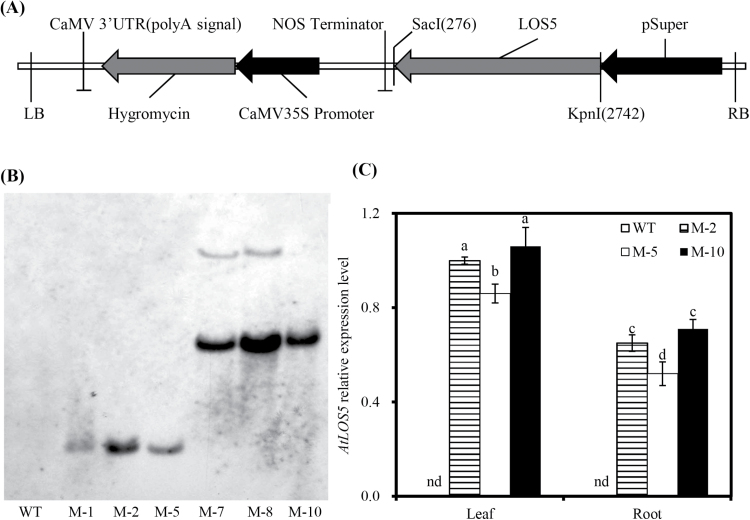
Molecular analysis of *AtLOS5*-expressing maize plants. (A) Schematic structure of the T-DNA region of the vector pCAMBIA1300-*AtLOS5.* LB, left T-DNA border; RB, right T-DNA border; pSuper, ‘super promoter’; Hygromycin, hygromycin phosphotransferase II gene; CaMV, *Cauliflower mosaic virus*. (B) Southern blot analysis of transgene copy number in T_3_ transgenic lines. Genomic DNA was digested with *Eco*RI, and the Hyg probe was used for hybridization. (C) The expression level of *AtLOS5* in the root and shoot of transgenic lines. RNA was extracted from transgenic and WT leaves. Values were the mean ±SD (*n*=3).

Transgenic *AtLOS5* maize (*Zea mays* L. cv. Zheng 58) was produced previously (Lu *et al.*, 2013). In brief, *AtLOS5* was cloned as an *Xba*I–*Kpn*I fragment downstream of the super promoter in modified pCAMBIA 1300, and the constitutive super promoter includes three copies of the octopine synthase enhancer in front of the manopine synthase promoter (Fig. 1A). The recombinant plasmid was introduced into *Agrobacterium tumefaciens* strain *EHA105* used to transform maize. Three transgenic lines (M-2, M-5, and M-10) with a single-copy insertion of *AtLOS5* were used in this study. Seeds of transgenic and WT plants were surface-sterilized for 10min with 75% (v/v) ethanol solution, rinsed with sterilized distilled water five times, and kept for 7 d at 25 °C in sand for germination. Residual embryo tissues were trimmed off and seedlings lightly fixed to holes of polyvinyl chloride board using sponge. After that, seedlings were transferred to 5 litre plastic containers (the outside of the container was wrapped in foil to block out light) containing aerated half-strength modified Hoagland’s nutrient solution for 4 d (acclimation treatment), and then the strength of the nutrient solution was increased to full strength. The full-strength nutrient solution had the following composition: 5.0mM CaCl_2_, 2.5mM Ca(NO_3_)_2_, 1.0mM K_2_SO_4_, 0.6mM MgSO_4_, 0.2mM KH_2_PO_4_, 2.0 μM MnSO_4_, 1.0 μM H_3_BO_4_, 0.5 μM ZnSO_4_, 0.3 μM CuSO_4_, 0.005 μM (NH_4_)_6_Mo_7_O_24_, and 200 μM Fe-EDTA. Subsequently, the nutrient solution was changed every 2 d. Eight plants per pot, two of each genotype, were grown in a climate chamber with a 14h photoperiod and a 25/30 °C night/day temperature cycle, 400 μmol m^−2^ s^−1^ irradiance, and relative humidity of 60%.

When plants reached the fourth fully expanded leaf (V_4_) stage, half were exposed to 100mM NaCl for 7 d, while the remainder continued to receive nutrient solution without added NaCl. Plant height and biomass were used to evaluate the salt tolerance of transgenic lines.

Healthy and uniform transgenic and WT seedlings were used for iso-osmotic NaCl and polyethlene glycol (PEG) treatments. The PEG used had a mean mol. wt of 6000kDa and a concentration of 147g l^−1^, and had the same osmotic strength (0.46MPa) as 100mM NaCl, as measured with a freezing point osmometer (Vapor 5520, Wescor Inc., UT, USA). Samples were harvested at 0, 6, 12, 24, 48, and 72h of treatments to analyse gene expression, and at 0, 1, 3, 5, and 7 d of treatments for measuring physiological and biochemical parameters. The harvested fresh samples were rinsed with sterilized distilled water, frozen in liquid nitrogen, and stored at −80 °C. To evaluate the growth rate and leaf water potential of maize exposed to salt stress, plants were exposed to 0, 25, 50, 75, and 100mM NaCl, and leaf water potential and plant fresh weight and dry weight were measured after 0, 1, 3, 5, and 7 d of treatments. The fresh weight and dry weight of the harvested samples were determined.

### PCR assay and Southern blotting analysis

Genomic DNA was isolated from expanding leaves of transgenic and WT plants with the trimethylamonium bromide protocol. The presence and integrity of the transgene were further confirmed by PCR analysis using *AtLOS5*-specific primers (5′-CCTGATGGCTCTTGGTTTGGCTAC-3′ and 5′-TTCCACTGACGACGGTTCCATTCC-3′). Southern blotting was performed with the digoxigenin (DIG)-labelled PCR-amplied gene fragment of the *AtLOS5* probe as described in the DIG System Manual (Roche Corporation, Basel, Switzerland). In brief, genomic DNA from T_3_ transgenic and WT plants was digested with the restriction enzyme *Eco*RI at 37 °C overnight. Digested DNA was separated on a 0.8% (w/v) agarose gel and blotted onto Hybond N^+^ nylon membrane. The membrane was hybridized with a DIG-labelled Hyg-specific probe (Hyg-313F 5′-CACGGCCTCCAGAAGAAGATGTT-3′ and Hyg-787R 5′-TGGGGAGTTTAGCGAGAGCCTGAC-3′) at 68 °C for 16h. The hybridized membrane was washed and detected according to the protocol of the DIG Nucleic Acid Detection Kit.

### RNA isolation and real-time quantitative PCR analysis

Total RNA was isolated from leaves and roots of transgenic and WT plants using TRIZOL^®^ reagent (Invitrogen, Carlsbad, CA, USA) and purified using Qiagen RNeasy columns (Qiagen, Hilden, Germany) according to the instructions of the manufacturer, and then reverse transcription was performed using Moloney murine leukemia virus (Invitrogen). Real-time quantitative RT-PCR was performed on a 7500 real-time PCR system (Applied Biosystems, CA, USA) using SYBR® Premix Ex Taq™ (Takara, Japan). The Primer Express program 3.0 (Applied Biosystems, Foster City, CA, USA) was used to design the primers for the genes chosen (Supplementary Table S1 available at *JXB* online), and the *Actin* gene was chosen as an internal control to normalize the data. According to the manufacturer’s protocol, a melt-curve analysis was performed to monitor primer–dimer formation and amplification of gene-specific products. The relative quantification method was used to evaluate quantitative variation between replicates.

### Analysis of water relation parameters

Leaf water potential was determined in the uppermost fully expanded leaves using a pressure chamber (Model 3000, Soil Moisture Equipment Corp., CA, USA). After excising the shoot inside the climate chamber, the shoot was immediately transferred to a humidity-saturated plastic box in low light. Leaf blades were cut 8cm (below the leaf tip) in length, rapidly sealed in the pressure chamber, and the leaf water potential was measured. During the measurements, the inner wall of the pressure chamber was lined with wet filter paper to reduce evaporative demand. Root water potential was measured following the same procedure. After that, leaf and root samples were frozen and thawed in a sealed cup, and the osmotic potential of the leaf and root was determined using a vapour pressure osmometer (Vapor 5520, Wescor Inc., UT, USA). Turgor potential was calculated as the difference between water potential and osmotic potential values.

A plant water status console (Model 3005F01, Soil Moisture Equipment Corp.) was used to determine *L*pr (Horie *et al.*, 2011). The intact root was sealed into a pressure chamber filled with either nutrient solution or solution supplemented with 100mM NaCl or iso-osmotic PEG. Initially, 0.12MPa pressure was applied to the pressure chamber with compressed N_2_ gas, then pressure was gradually reduced by 0.02MPa each time (5min) until it reached 0.02MPa. The osmolarities of the exuded sap and nutrient solution were determined using a vapour pressure osmometer (Vapor 5520, Wescor Inc.). The sap flow rate of individual samples (*J*v) was determined by measuring the sap weight per unit time. *J* was calculated as *J*v/∆Ψ, where ∆Ψ was the osmotic potential difference between the exuded sap and the nutrient solution. The root surface area of individual samples (*A*) was measured using the WinRHIZO system (version 5.0, Regent Instruments Inc., Quebec, Canada). *J*/*A* was plotted against *P*, and the regression line was determined between *P*=0.02MPa and *P*=0.12MPa. *L*pr was calculated as the slope of the regression line.

### Analysis of physiological and biochemical parameters

Membrane damage was assayed by measuring electrolyte leakage from leaf discs as described by Verslues *et al.* (2006). Proline concentrations were determined by the sulphonic acid method as previously described in Chen *et al.* (2007). Extraction and purification of ABA were carried out as previously described (Yang *et al.*, 2001), and ABA concentrations were determined by an indirect ELISA technique. The AO (EC 1.2.3.1) activity was measured by native PAGE as described by Porch *et al.* (2006). Native PAGE was carried out with a Protein IIxi Cell (JunYi, Beijing, China). Subsequently, gels were scanned in a Bio-Rad ChemiDoc SRS (Bio-Rad, CA, USA).

### Determination of Na^+^ and K^+^ concentration

Seedlings were harvested from the different treatments, and their roots were immersed in deionized water for 10min. Seedlings were oven-dried at 80 °C for measurement of biomass. For ion content measurements, dried material of separated roots and shoots was milled to a powder and extracted with 1.0M HCl for 24h by shaking for 30min at 30 °C. Na^+^ and K^+^ concentrations were determined in diluted extracts using an atomic absorption spectrophotometer (SpectAA-50/55, Varian, Australia).

### Measurements of net Na^+^, K^+^, and H^+^ flux with NMT

Net Na^+^, H^+^, and K^+^ fluxes were measured using the non-invasive micro-test technology (NMT) (NMT system BIO-IM, LLC, MA, USA). The principle behind this method and the instrument used were described previously (Sun *et al.*, 2009). After exposure to 100mM NaCl or iso-osmotic PEG for 2 d, root segments with 2–3cm apices were sampled for ion flux measurements. Roots were rinsed with redistilled water and immediately incubated in the measuring solution to equilibrate for 30min. After that, roots were transferred to a measuring chamber containing 10–15ml of a fresh measuring solution. After roots were immobilized on the bottom, ion flux measurements were started from the apex and continued along the root axis until 1500 μm. Then the measuring site was selected at 400 μm from the root apex (located within the elongation zone of the root), in which a vigorous flux of K^+^, Na^+^, and H^+^ was usually observed. Ions were monitored in the following measuring solutions: (i) Na^+^, 0.1mM KCl, 0.1mM CaCl_2_, 0.1mM MgCl_2_, 0.5mM NaCl, 0.3mM MES, 0.2mM Na_2_SO_4_, pH 6.0 adjusted with choline and HCl; (ii) K^+^, 0.1mM KCl, 0.1mM CaCl_2_, 0.1mM MgCl_2_, 0.3mM MES, 0.2mM Na_2_SO_4_, pH 6.0 adjusted with TRIS and HCl; and (iii) H^+^, 0.1mM KCl, 0.1mM CaCl_2_, 0.1mM MgCl_2_, 0.5mM NaCl, 0.2mM Na_2_SO_4_, pH 6.0 adjusted with NaOH and HCl. Net fluxes were calculated using JCal V3.2.2 (xuyue.net).

The measurements of the effects of salt shock and ion transporter inhibitors on ion fluxes were described by Sun *et al.* (2009). Transient K^+^ flux kinetics in response to 100mM NaCl were detected in root tips from transgenic and WT plants. A steady-state K^+^ flux was recorded (5–6min) prior to salt shock, then NaCl stock (1M) was slowly added to the measuring solution until the final NaCl concentration in the buffer reached 100mM. After that, K^+^ flux recording was restarted and continued for a further 20–30min. Data measured during the first 2–3min were discarded due to the diffusion effects of stock addition (Sun *et al.*, 2009). In addition, roots were incubated in measuring solution with 500 μM sodium orthovanadate (PM H^+^-ATPase inhibitor), 100 μM amiloride (Na^+^/H^+^ antiporter inhibitor), or 50mM TEA (K^+^ channel blocker) for 30min. The measuring solutions were removed slowly with a pipette, and 10ml of fresh solution was slowly added to the measuring chamber. K^+^, Na^+^, and H^+^ fluxes were scanned at a location 400 μM from the root apex.

### Statistical analysis

Results are based on two independent experiments with at least three replicate samples from transgenic and WT plants in each treatment. All data were statistically analysed using the SPSS package (version 13.0; SPSS Inc., IL, USA), and the mean and SD of each treatment as well as least significant difference (LSD; *P*<0 .05 and *P*<0.01) for each set of corresponding data were calculated.

## Results

### Generation and identification of transgenic maize plants overexpressing *AtLOS5*

Sixteen independent maize transgenic lines were generated, and the transformation of six T_2_ plants was confirmed by RT-PCR analysis (Supplementary Fig. S1 at *JXB* online). All independent T_3_ transgenic plants were exposed to salt stress, and six independent representative transgenic plants showed higher salt resistance than the WT based on leaf water status and biomass (data not shown). Copy numbers and integration events of the transgene in transgenic plants were determined by genomic Southern blots, which revealed that all the lines with higher salt resistance were independent and that the copy numbers of the corresponding transgene were either one (M-1, M-2, M-5, and M-10) or two (M-7 and M-8; Fig. 1B). *AtLOS5* was expressed in both shoot and root tissues (Fig. 1C), and three homozygous transgenic lines (M-2, M-5, and M-10) with integration of a single-copy transgene were selected for further analysis.

### *AtLOS5* overexpression improved plant growth in maize plants exposed to salt stress

Upon treatment with 100mM NaCl for 7 d, transgenic plants were taller than WT plants (Table 1). Transgenic plants produced more dry mass than the WT, and, in both cases, the difference in root biomass was markedly greater than that of shoot biomass. For example, transgenic lines M-2, M-5, and M-10 produced 23.8, 20.4, and 27.3% more dry shoot mass and 28.8, 32.7, and 40.3% more dry root mass than the WT, respectively. After salt stress, the root/shoot ratio of M-2, M-5, and M-10 was significantly higher than that of the WT. Although the relative growth rate decreased with time at all salt levels, transgenic plants showed higher relative growth rates than WT plants when exposed to salt (Supplementary Fig. S2 at *JXB* online). However, plant height, dry mass, relative growth rate, and root/shoot ratio under control conditions were similar for transgenic and WT plants.

**Table 1.** Plant height and biomass assay in transgenic and WT maize under salt stress

Lines	Plant height (cm)		Shoot DW (g per plant)		Root DW (g per plant)		Root/shoot ratio	
	Control	NaCl	Control	NaCl	Control	NaCl	Control	NaCl
WT	81.12 a	56.03 c	2.783 a	1.611 c	0.598 a	0.392 c	0.214 a	0.243 c
M-2	80.00 a	60.30 b	2.780 a	1.995 b	0.612 a	0.505 b	0.220 a	0.253 b
M-5	79.62 a	63.24 a	2.846 a	1.940 b	0.624 a	0.520 b	0.219 a	0.268 a
M-10	79.41 a	63.56 a	2.753 a	2.050 a	0.616 a	0.550 a	0.224 a	0.269 a

NaCl, nutrient solution to which 100mM NaCl was added for 7 d. Control, nutrient solution without added NaCl.

Values are the mean ±SD (*n*=10), and different letters indicate significant differences (*P*<0.05) among the treatments.

**Fig. 2.**
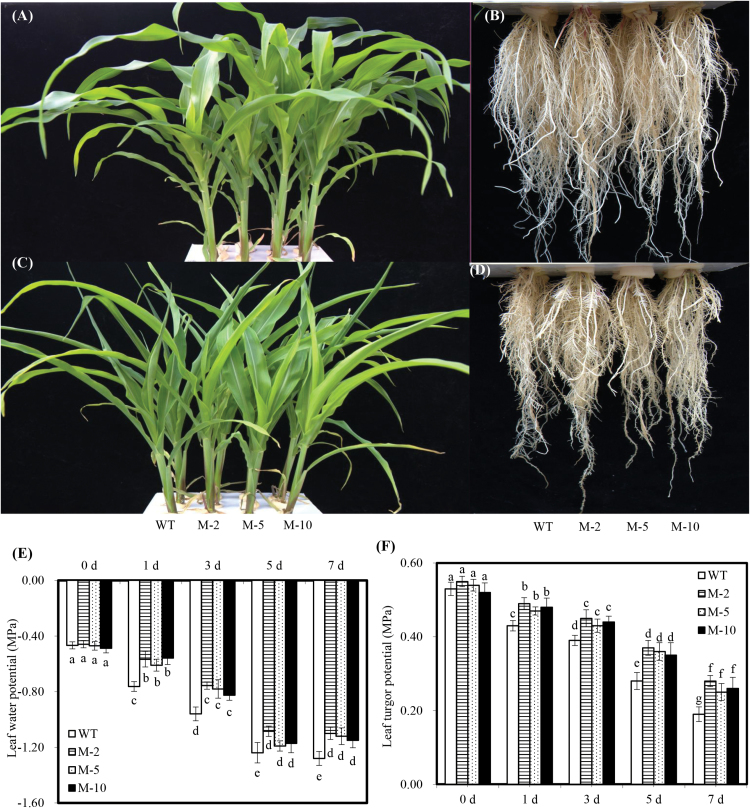
Phenotype and leaf water status of transgenic *AtLOS5* and WT maize plants under salt stress and control conditions. (A and B) Leaf and root phenotype of transgenic and WT plants under control conditions. (C and D) Leaf and root phenotype of transgenic and WT plants exposed to 100mM NaCl for 7 d. (E and F) Leaf water potential and turgor potential of transgenic and WT plants subjected to 100mM NaCl for 7 d. Control condition denotes nutrient solution without added NaCl. Values are the mean ±SD (*n*=6), and different letters indicate significant differences (*P*<0.05) among the treatments.

No apparent difference was observed between transgenic and WT plants with regard to their growth and development under control conditions (Fig. 2A, B). After 7 d of salt stress, the leaves of WT plants exhibited moderate wilting, but slight leaf wilting was observed in transgenic plants (Fig. 2C). Moreover, transgenic plants exhibited more robust root systems compared with the WT under salt stress (Fig. 2D). After imposing salt stress, transgenic plants had a higher leaf water potential and turgor compared with WT plants, but the leaf water potential of transgenic plants was similar to that of the WT under control conditions (Fig. 2E, F). In addition, leaf water potential decreased as salt concentration increased, and increasing the salt concentration progressively decreased leaf water potential over time in both transgenic and WT plants; thus, a significant positive relationship was observed between relative growth rate and leaf water potential (Supplementary Fig. S3 at *JXB* online).

### *AtLOS5* overexpression regulated ABA biosynthesis to enhance ABA accumulation

**Fig. 3.**
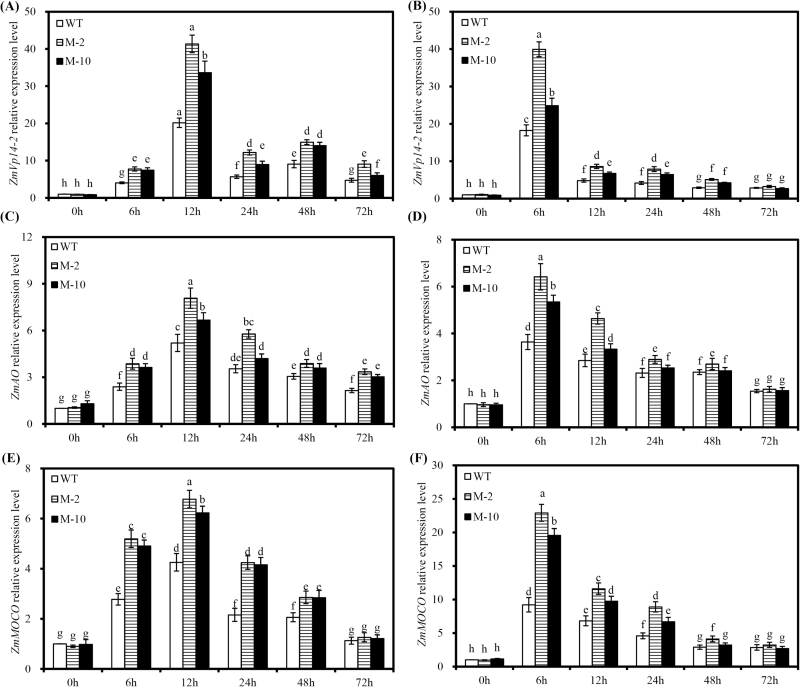
The expression of *ZmVp14-2*, *ZmAO*, and *ZmMOCO* in leaves and roots of transgenic and WT plants exposed to 100mM NaCl. (A), (C), and (E) The expression of *ZmVp14-2*, *ZmAO*, and *ZmMOCO* , respectively, in leaves of transgenic and WT plants. (B), (D), and (F) The expression of *ZmVp14-2*, *ZmAO*, and *ZmMOCO* , respectively, in roots of transgenic and WT plants. The expression level of transgenic and WT plants is shown relative to the expression of WT plants grown in nutrient solution without added NaCl at 0h. Values are the mean ±SD (*n*=3), and different letters indicate significant differences (*P*<0.05) among the treatments.

There was no significant difference in expression of *ZmVp14-2* between transgenic and WT plants under control conditions. Salt stress significantly enhanced leaf and root expression of *ZmVP14-2* in transgenic plants compared with WT plants. Salt stress rapidly induced foliar *ZmVp14-2* expression (within 6h), which peaked at 12h and then decreased thereafter. Induction of *ZmVp14-2* in the root due to salt stress reached a maximum at 6h and then decreased (Fig. 3A, B). Similar patterns were observed for the expression levels of *ZmAO* and *ZmMOCO* in roots and leaves of transgenic and WT plants under salt stress. Compared with WT plants, the expression levels of these genes in transgenic M-2 and M-10 plants were significantly up-regulated at 6–48h in leaves and at 6–24h in roots (Fig. 3C–F).

**Fig. 4.**
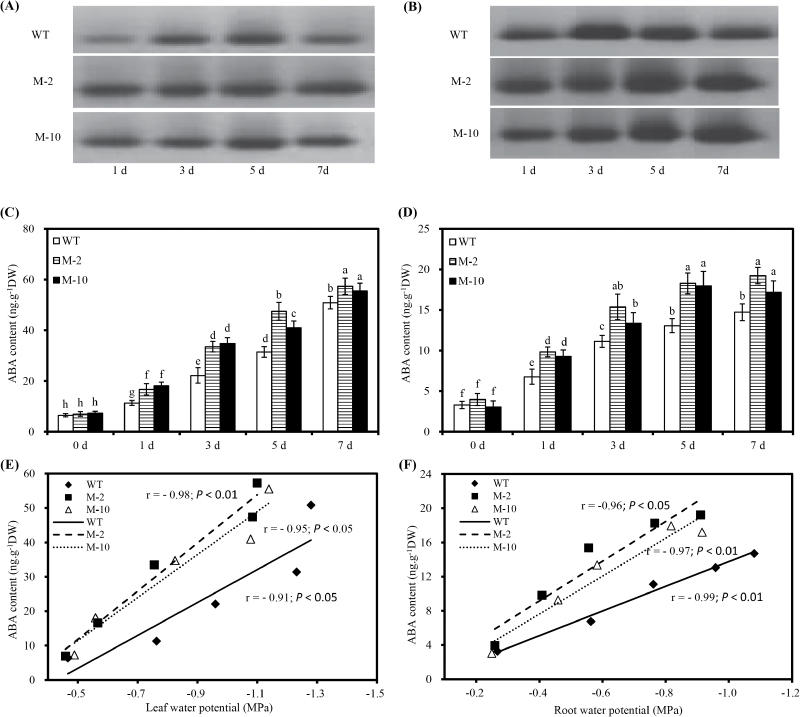
AO activity, ABA concentrations, and the relationship between ABA concentration and water potential in leaves and roots of transgenic and WT plants under salt stress. (A and B) Native PAGE assay for AO activity from transgenic maize and WT leaf and root extracts under 100mM NaCl, respectively. (C and D) Changes of ABA concentrations in leaf and root of transgenic and WT maize exposed to salt stress, respectively. (E and F) The relationship between ABA concentration and water potential in leaf and root of transgenic and WT maize. *r* indicates the correlation coefficient, and the *P*-value indicates significant differences at the 0.05 or 0.01 levels. Values are the mean ±SD (*n*=6), and different letters indicate significant differences (*P*<0.05) among the treatments.

Salt stress increased foliar AO activities of transgenic plants compared with WT plants (Fig. 4A). Also, root AO activity of the transgenic lines M-2 and M-10 was markedly greater than that of the WT at 1, 5, and 7 d after salt stress (Fig. 4B). Root and leaf ABA concentrations increased continuously with time under salt stress, with concentrations significantly higher in transgenic than in WT plants (Fig. 4C, D). Under control conditions, ABA concentrations were similar between transgenic and WT plants. Both leaf and root ABA concentrations significantly increased with the decline in leaf and root water potential, respectively, with transgenic plants having a higher ABA concentration at a similar water potential (Fig. 4E, F).

### *AtLOS5* overexpression led to accumulation of higher K^+^ under salt stress

**Fig. 5.**
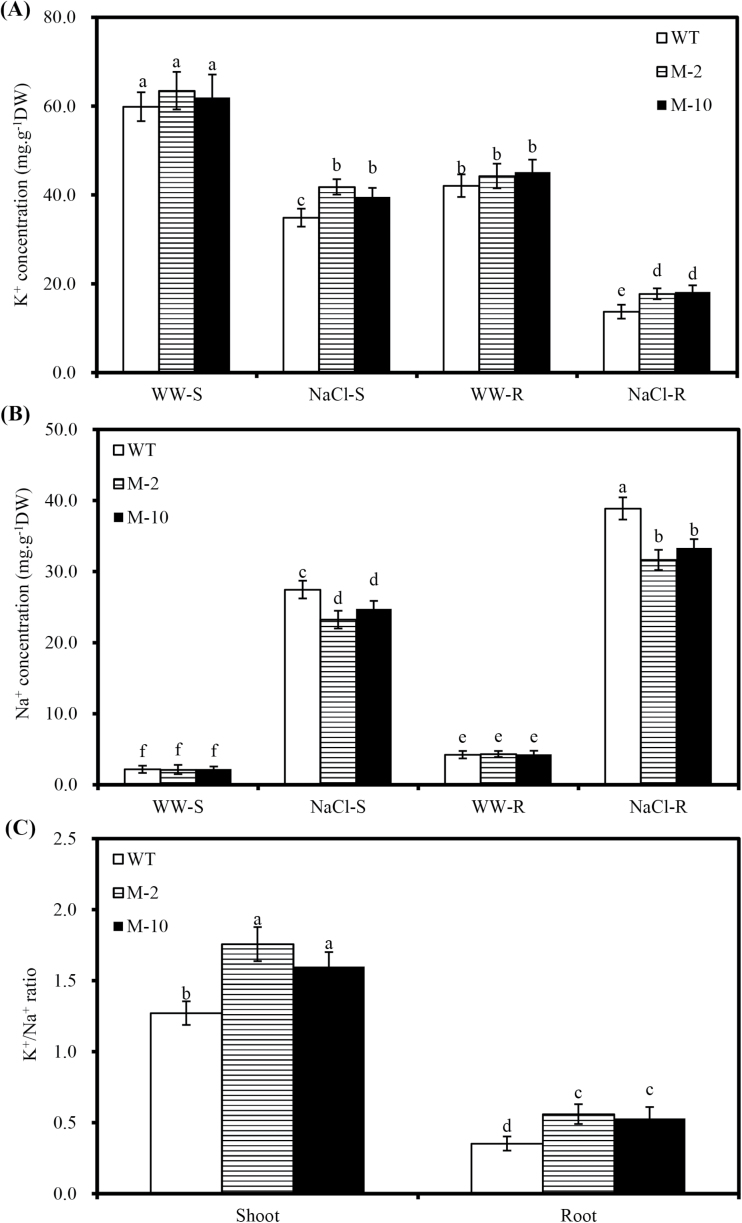
Concentrations of K^+^ and Na^+^ in shoots and roots of transgenic and WT plants under salt stress. (A) K^+^ concentrations in the shoots and roots of transgenic and WT plants treated with 100mM NaCl for 7 d. WW-S and NaCl-S indicate the shoots of plants under nutrient solution with 0 and 100mM NaCl solution added. WW-R and NaCl-R indicate the roots of plants under nutrient solution with 0 and 100mM NaCl added. (B and C) Na^+^ concentrations and K^+^/Na^+^ ratio in the shoots and roots of transgenic and WT maize plants exposed to 100mM NaCl for 7 d. Values are the mean ±SD (*n*=5), and different letters indicate significant differences (*P*<0.05) among the treatments.

Under salt stress, shoot K^+^ concentrations of M-2 and M-10 were 19.8% and 13.5% higher than those of WT plants, while root K^+^ concentrations were 29.% and 32.9% higher, respectively (Fig. 5A). In contrast, shoot Na^+^ concentrations of M-2 and M-10 were 18.2% and 10.9% lower than those of WT plants, while root Na^+^ concentrations were 22.9% and 16.6% lower, respectively (Fig. 5B). However, there were no obvious differences in Na^+^ and K^+^ concentrations in transgenic and WT plants under control conditions. When exposed to salt stress, transgenic lines M-2 and M-10 had higher K^+^/Na^+^ ratios than WT plants (Fig. 5C). For example, the K^+^/Na^+^ ratio of M-2 and M-10 was 38.2% and 25.9% higher in the shoot, and 59.1% and 50.5% higher in the root compared with salinized WT plants.

### *AtLOS5* overexpression changed root K^+^, Na^+^, and H^+^ fluxes and modulated salt-induced gene expression in response to salt stress and mannitol

**Fig. 6.**
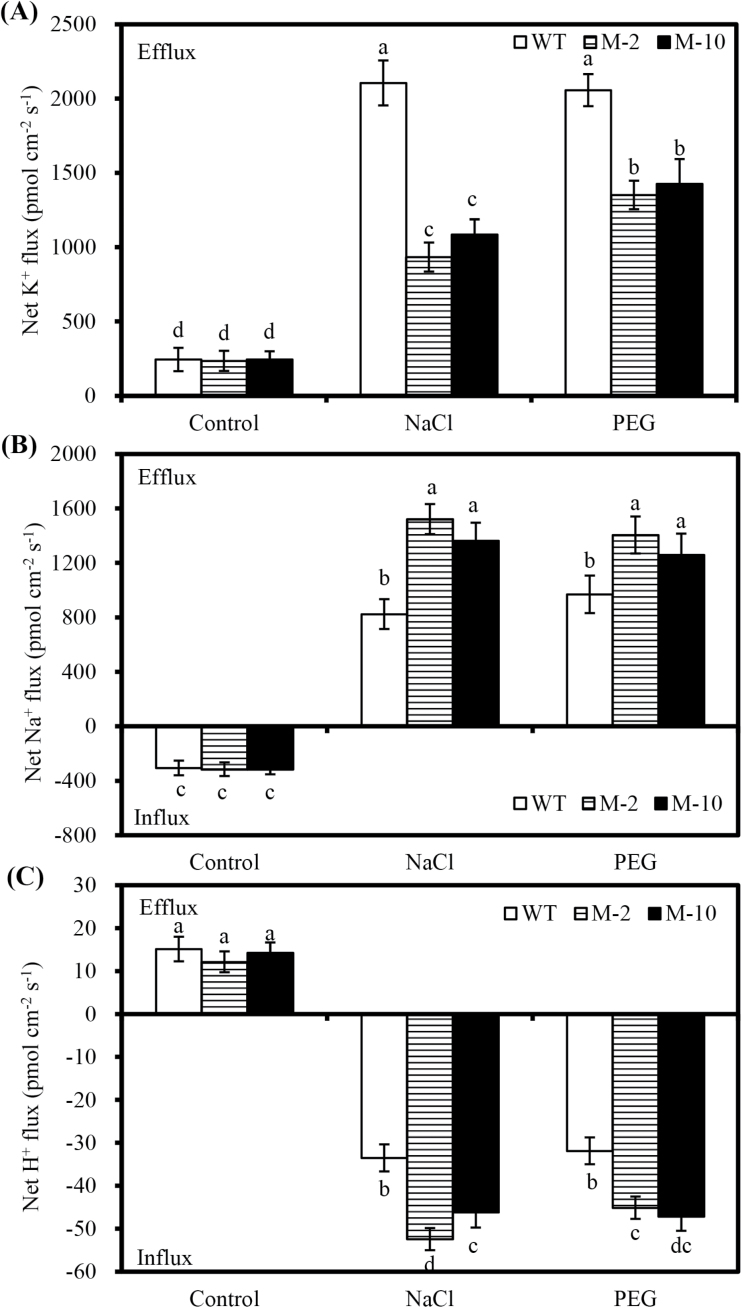
Net K^+^ (A), Na^+^ (B), and H^+^ (C) fluxes in roots of transgenic and WT plants exposed to 100mM NaCl or iso-osmotic PEG treatments for 2 d. Control denotes nutrient solution without added NaCl. Values are the mean ±SD (*n*=3), and different letters indicate significant differences (*P*<0.05) among the treatments.

NaCl induced a marked K^+^ efflux in transgenic and WT roots, but the net K^+^ effluxes in transgenic roots were significantly lower than those in the WT under salt stress (Fig. 6A). An iso-osmotic stress caused by PEG also markedly promoted the net K^+^ efflux, and net K^+^ effluxes in transgenic roots were markedly less than in the WT under iso-osmotic PEG treatment. No obvious differences in the net K^+^ effluxes were observed between transgenic and WT plants under control conditions.

There was a net Na^+^ influx in transgenic and WT roots under control conditions, but net Na^+^ flux was reversed to an efflux when they were treated with 100mM NaCl or iso-osmotic PEG (Fig. 6B). Net Na^+^ effluxes in transgenic roots were markedly greater than in WT roots exposed to salt stress, whereas net Na^+^ influxes were similar between transgenic and WT plants under control conditions. Similar to NaCl treatment, net Na^+^ effluxes in transgenic roots were higher than in WT roots under iso-osmotic PEG treatment. In contrast, a net H^+^ efflux in transgenic and WT roots was observed under control conditions, but net H^+^ influx was exhibited when subjected to 100mM NaCl or iso-osmotic PEG (Fig. 6C). Net H^+^ influxes in transgenic roots were significantly higher than in WT roots under 100mM NaCl or iso-osmotic PEG, but net H^+^ effluxes showed no obvious difference in transgenic and WT roots under control conditions.

**Fig. 7.**
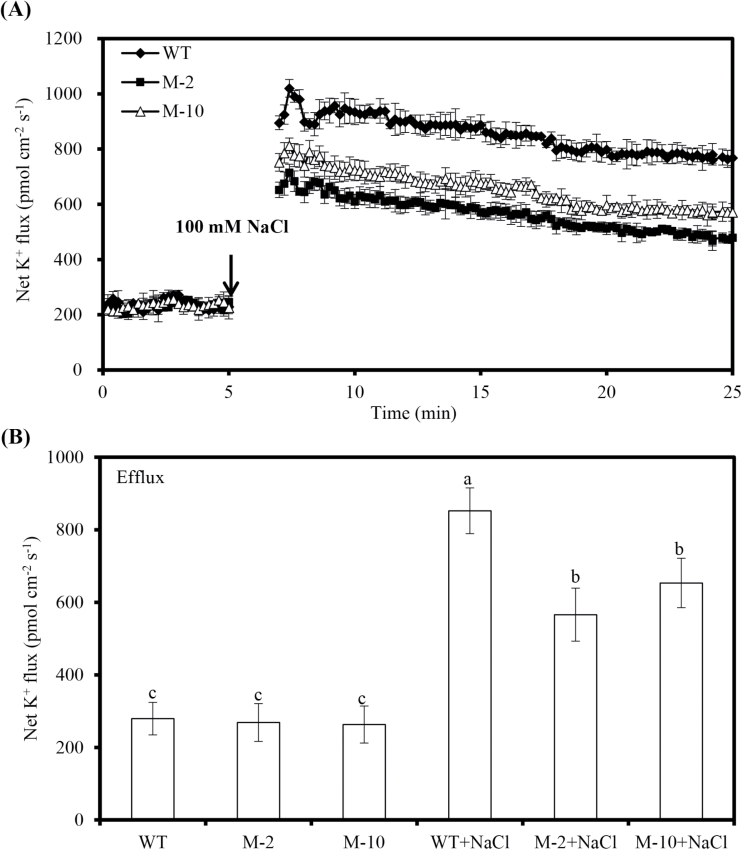
Transient K^+^ kinetics of salt shock (100mM NaCl) in transgenic and WT roots. (A) Transient K^+^ flux kinetics in response to100mM NaCl. Prior to the salt shock, steady K^+^ fluxes of roots were detected for ~5min. Values are the mean ±SD (*n*=3). (B) The mean rate of K^+^ flux in transgenic and WT roots during the period of salt shock. Values are the mean ±SD (*n*=3), and different letters indicate significant differences (*P*<0.05) among the treatments.

**Fig. 8.**
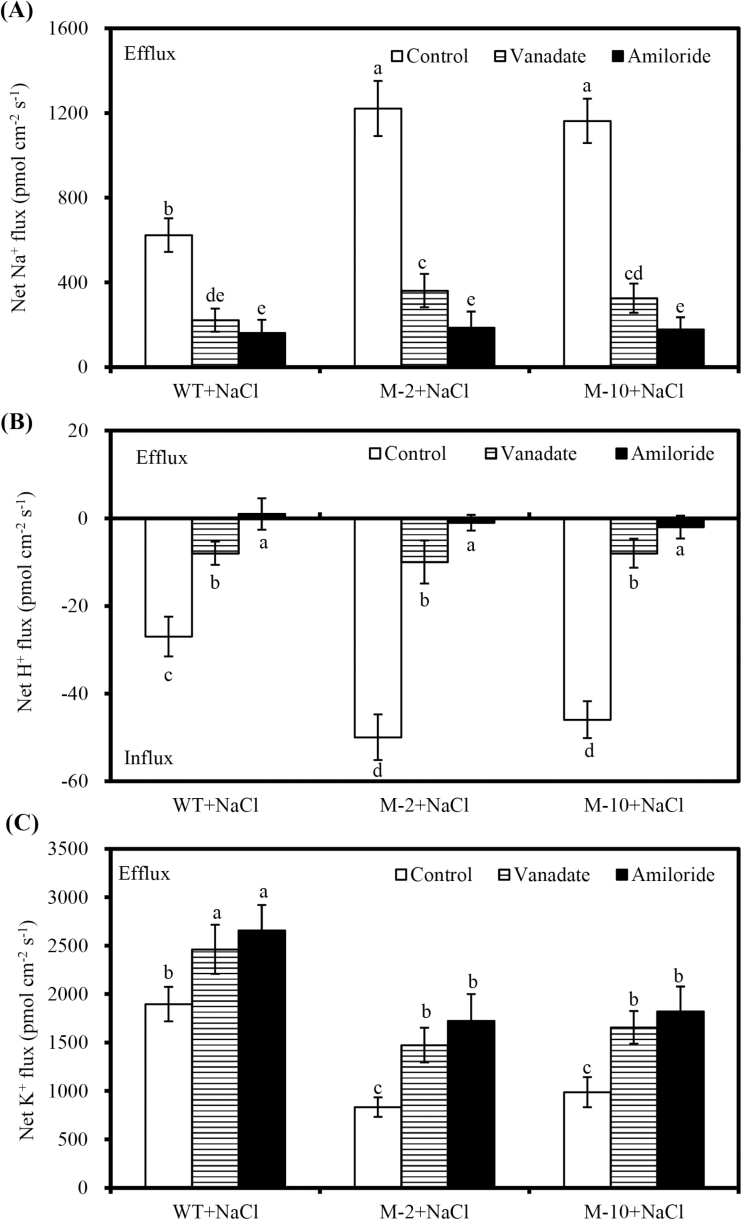
Effects of sodium orthovanadate and amiloride on the net Na^+^ (A), H^+^ (B), and K^+^ (C) fluxes in transgenic and WT roots exposed to 100mM NaCl treatment for 2 d. Control denotes NaCl-treated roots without sodium orthovanadate or amiloride treatments. Values are the mean ±SD (*n*=3), and different letters indicate significant differences (*P*<0.05) among the treatments.

NaCl shock induced a significant net K^+^ efflux in transgenic and WT roots, and the efflux decreased over time (Fig. 7A). However, net K^+^ effluxes of transgenic plants were much lower than those of the WT (Fig. 7B). In addition, the PM transport inhibitors amiloride or sodium orthovanadate significantly inhibited the NaCl-induced net Na^+^ efflux and H^+^ influx in roots (Fig. 8A, B). In contrast, net K^+^ effluxes in transgenic and WT roots were markedly elevated by amiloride or sodium orthovanadate, and the increase of net K^+^ effluxes in transgenic roots was greater than in WT roots (Fig. 8C).

**Fig. 9.**
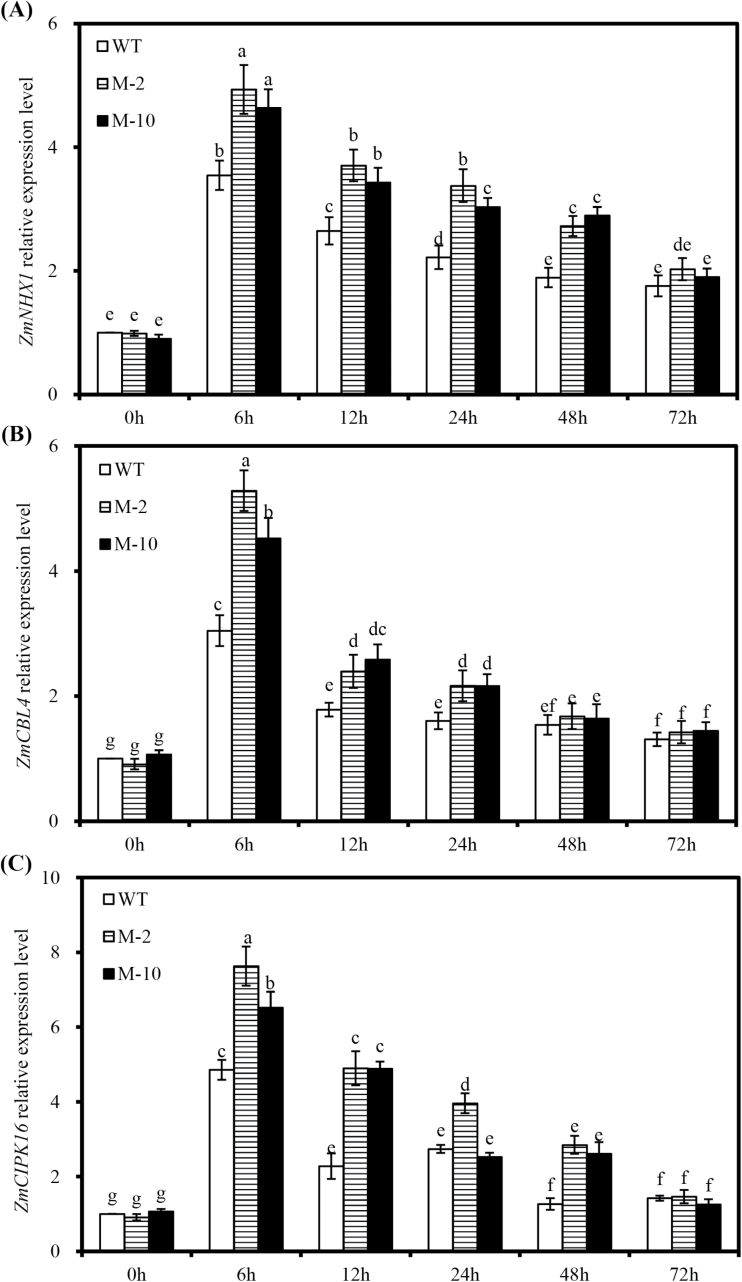
Expression patterns of *ZmNHX1* (A), *ZmCBL4* (B), and Z*mCIPK16* (C) in transgenic and WT roots under salt stress. The gene expression levels of transgenic and WT plants are shown relative to the expression of WT plants grown under nutrient solution without added NaCl at 0h. Values are the mean ±SD (*n*=3), and different letters indicate significant differences (*P*<0.05) among the treatments.

NaCl significantly induced the expression of *ZmNHX1* in transgenic and WT roots under salt stress (Fig. 9A), which peaked at 6h, and then decreased at 12–72h after salt stress. Moreover, *AtLOS5* overexpression significantly enhanced the expression of *ZmNHX1* in transgenic roots compared with WT roots at 6–48h after salt stress. The expression of *ZmNHX1* was similar between transgenic and WT plants under control conditions. Similar patterns were observed for the expression levels of *ZmCBL4* and *ZmCIPK16* in transgenic and WT roots under salt stress (Fig. 9B, C).

### *AtLOS5* overexpression alleviated the reduction of root hydraulic conductivity and regulated the expression of *ZmPIP* genes under salt stress

**Fig. 10.**
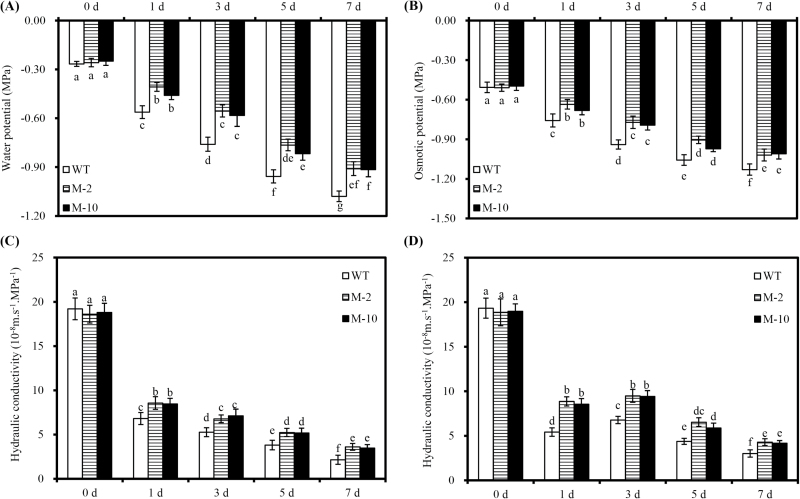
Root water potential, osmotic potential, and hydraulic conductivity in transgenic and WT plants subjected to 100mM NaCl or iso-osmotic PEG for 7 d. (A and B) Root water potential and osmotic potential of transgenic and WT plants subjected to 100mM NaCl for 7 d. (C and D) Root hydraulic conductivity of transgenic and WT plants exposed to 100mM NaCl or iso-osmotic PEG for 7 d, respectively. Values are the mean ±SD (*n*=6), and different letters indicate significant differences (*P*<0.05) among the treatments.

Root water potential was strongly reduced after 1 d of salt stress, and continued to decline with time (Fig. 10A). However, the root water potential of transgenic lines was significantly higher than that in WT plants. Similar patterns were found for root osmotic potential (Fig. 10B).

There were no obvious differences in *L*pr between transgenic and WT roots under control conditions. Salt stress dramatically reduced *L*pr, but transgenic plants showed significantly greater *L*pr than WT plants (Fig. 10C). Iso-osmotic PEG treatment evoked nearly identical reductions in *L*pr to the corresponding NaCl treatment, and again transgenic plants showed much higher *L*pr than WT plants (Fig. 10D).

**Fig. 11.**
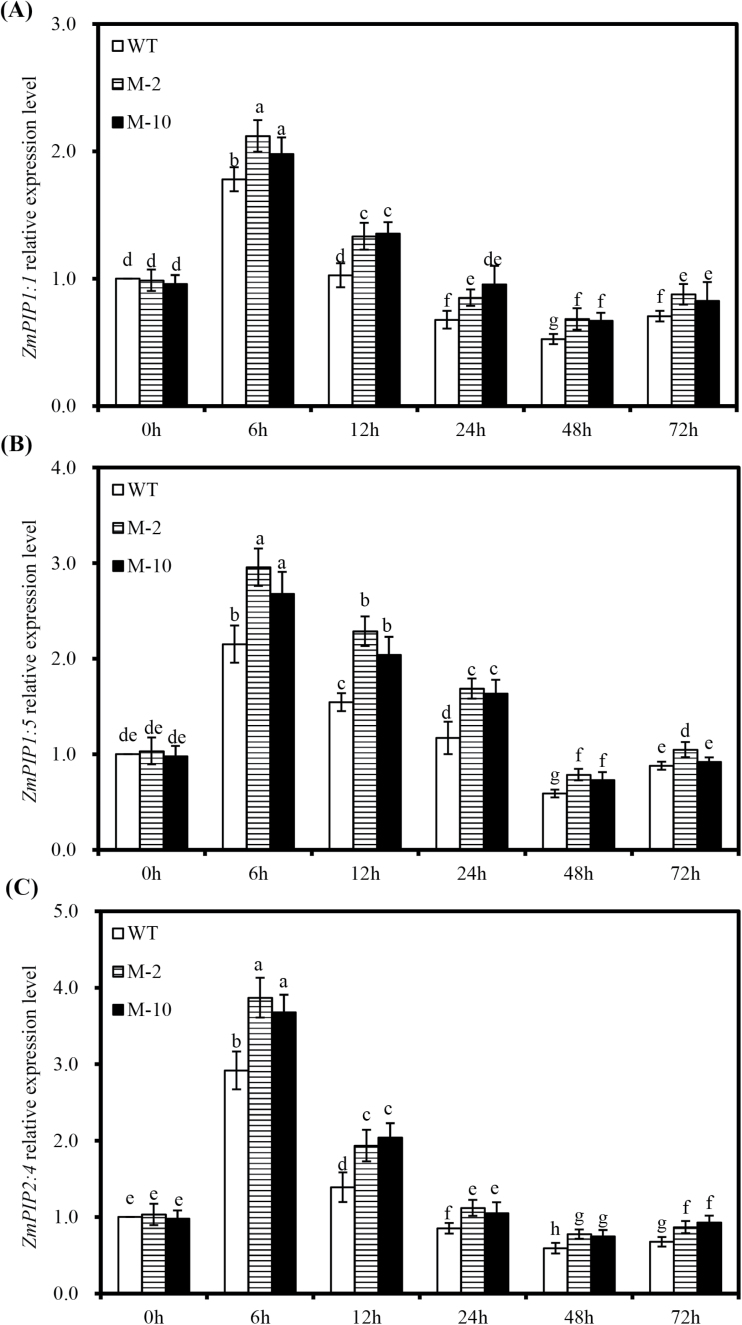
Expression patterns of *ZmPIP1:1* (A), *ZmPIP1:5* (B), and *ZmPIP2:4* (C) in transgenic and WT roots under salt stress. The gene expression levels of transgenic and WT plants are shown relative to the expression of WT plants grown under nutrient solution without added NaCl at 0h. Values are the mean ± SD (*n*=3), and different letters indicate significant differences (*P*<0.05) among the treatments.

A transient induction of *ZmPIP1:1*, *ZmPIP1:5*, and *ZmPIP2:4* expression was observed at 6h after salt stress, which then decreased thereafter (Fig. 11A–C). Moreover, *AtLOS5* overexpression significantly enhanced the expression of *ZmPIP1:1*, *ZmPIP1:5*, and *ZmPIP2:4* in transgenic roots compared with WT roots under salt stress. The expression levels of *ZmPIP1:1*, *ZmPIP1:5*, and *ZmPIP2:4* were similar between transgenic and WT plants under control conditions.

## Discussion

Salt stress induced a rapid and massive accumulation of ABA in plant tissues, which is very important for the perception of salt signal or the initial triggering of ABA accumulation (Zhang *et al.*, 2006). Introducing a single gene in the ABA biosynthesis pathway can improve plant salt resistance. For example, expression of the *NCED* gene in tobacco and creeping bentgrass activated ABA biosynthesis and enhanced salt tolerance (Aswath *et al.*, 2005; Zhang *et al.*, 2009). Overexpressing *ABA1* in Arabidopsis promoted ABA accumulation and improved resistance to salt stress (Park *et al.*, 2008). In this study, *AtLOS5* overexpression in maize decreased leaf wilting and enhanced plant height and biomass accumulation under salt stress. Similarly, sweetpotato plants expressing *LOS5* had enhanced salt tolerance, higher ABA concentrations, and higher antioxidant enzyme activities (Gao *et al.*, 2011). Nevertheless, our study further explored the effect of *AtLOS5* overexpression on ABA biosynthesis, intracellular K^+^ and Na^+^ homeostasis, and water uptake.

Under salt stress, *AtLOS5* overexpression up-regulated *ZmVp14-2*, *ZmMOCO*, and *ZmAO* expression and increased AO activities. These results were consistent with Xiong *et al.* (2002), who found that the initial accumulation of ABA from overexpressing one ABA biosynthesis gene stimulates the expression of other ABA biosynthesis genes, which then resulted in a co-ordinated increase in *de novo* ABA biosynthesis. However, our results showed that AO activity and ABA concentration showed no obvious difference between transgenic and WT plants under control conditions, although *AtLOS5* transcription was driven by a constitutive super promoter. A sulphurylated MoCo formed by the catalytic activity of *AtLOS5*-encoded MoCo sulphurase is also required by xanthine oxidase (XHD) for catalytic activity involved in degradation of purines (Xiong *et al.*, 2001; Porch *et al.*, 2006). Overexpressing *AtLOS5* significantly increased *ZmMOCO* expression under salt stress, but did not enhance *ZmMOCO* expression under control conditions, suggesting a positive feedback by ABA or salt stress. Constitutive expression of *AtLOS5* could not increase ABA accumulation under control conditions, but might be involved in regulating XHD.

Plant salt resistance is determined not only by the ability to restrain cytosolic Na^+^ accumulation, but also by maintaining a high cytosolic K^+^/Na^+^ ratio (Shabala and Cuin, 2008; Anschütz *et al.*, 2014). *AtLOS5* overexpression significantly increased K^+^ accumulation and decreased Na^+^ accumulation in the root and shoot of transgenic plants under salt stress, while the K^+^/Na^+^ ratio was much greater in transgenic plants (Fig. 5). ABA can promote the uptake of K^+^ in xylem sap and increase K^+^ ion channel activity, thereby enhancing K^+^ accumulation in plants (Chen *et al.*, 2006). These findings suggested that *AtLOS5* overexpression enhanced plant salt resistance through improved ABA accumulation to regulate intracellular K^+^ and Na^+^ homeostasis in order to maintain plant growth under salt stress.

Appropriate regulation of ion flux is necessary for cells to restrict the accumulation of toxic ions and accumulate essential ions under salt stress (Anschütz *et al.*, 2014). Under salt stress, H^+^ influx accompanied by Na^+^ efflux is observed in cotton (Kong *et al.*, 2012), Arabidopsis (Shabala *et al.*, 2005), and poplar (Sun *et al.*, 2009). Similarly, net Na^+^ efflux and H^+^ influx were obtained in maize roots exposed to salt stress, whereas net Na^+^ influx and H^+^ efflux were observed under control conditions. Moreover, *AtLOS5* overexpression markedly enhanced net Na^+^ efflux and H^+^ influx in transgenic roots compared with WT roots under salt stress (Fig. 6). Similarly, salt-tolerant poplar plants exhibited greater net Na^+^ efflux and H^+^ influx than salt-sensitive plants under NaCl stress (Sun *et al.*, 2009). Plants can use Na^+^/H^+^ antiporters localized in the vacuolar and plasma membranes to transport Na^+^ into the vacuolar lumen or out of the cell under salt stress (Shabala and Cuin, 2008). The activation of Na^+^/H^+^ antiporters by salt stress is positively correlated with salinity tolerance, and this effect in salt-tolerant plants is stronger than that of salt-sensitive plants (Chen *et al.*, 2007; Sun *et al.*, 2009). In this study, transgenic roots exhibited an evident increase in net H^+^ influx and a corresponding efflux of Na^+^ under salt stress, but the PM transport inhibitors amiloride and sodium orthovanadate simultaneously decreased Na^+^ efflux and H^+^ influx, which indicated that the Na^+^ extrusion in transgenic roots was mainly the result of an active Na^+^/H^+^ antiport across the PM.

The key role of N^+^/H^+^ antiport genes such as *AtSOS1* and *AtNHX1* has been explored by generation of salt-tolerant transgenic plants through overexpression of these genes in different species (Shi *et al.*, 2002; He *et al.*, 2005). Moreover, the transcript levels of *NHX* genes are up-regulated by ABA or osmotic stress (Shi and Zhu, 2002). The expression of *ZmNHX1* was remarkably induced by salt stress, and *AtLOS5* overexpression significantly increased *ZmNHX1* expression in transgenic roots compared with WT roots under salt stress (Fig. 9). Similarly, salt-tolerant hybrids exhibited a significant up-regulation of *ZmNHX1* in maize leaves compared with salt-sensitive hybrids subjected to salt stress (Pitann *et al.*, 2013). The AtSOS3 protein is required for maintaining K^+^ and Na^+^ homeostasis and salt tolerance in Arabidopsis (Qiu *et al.*, 2004). Wang *et al.* (2007) reported that overexpression *ZmCBL4*, a functional homologue of *AtSOS3*, can maintain low Na^+^ levels and high K^+^ levels in transgenic Arabidopsis seedlings under salt stress, and that *ZmCBL4* expression is up-regulated by ABA. Additionally, *ZmCIPK16* encodes a SOS2-like protein kinase, and overexpression of *ZmCIPK16* in the Arabidopsis *sos2* mutant induces *SOS1* expression and enhances salt tolerance, and *ZmCIPK16* is strongly induced by ABA and salt stress (Zhao *et al.*, 2009). We showed here that the expression levels of *ZmCBL4* and *ZmCIPK16* were significantly up-regulated by salt stress, and *AtLOS5* overexpression could remarkably enhance the expression of *ZmCBL4* and *ZmCIPK16* in transgenic roots compared with WT roots (Fig. 9). Combined with the analysis of *ZmNHX1* expression, transgenic maize plants had a high cytosolic K^+^/Na^+^ ratio through regulating ABA biosynthesis to activate the expression of K^+^ and Na^+^ transporters under salt stress.

K^+^ homeostasis plays a crucial role in the salt adaptation of plant cells (Shabala and Cuin, 2008; Anschütz *et al.*, 2014). Many investigations suggested that salt-tolerant cultivars show much lower K^+^ efflux compared with salt-sensitive cultivars under salt stress (Sun *et al.*, 2009; Shabala and Pottosin, 2014). Similar results were also observed in this study; NaCl-induced root K^+^ efflux was lower in *AtLOS5*-overexpressing plants than in the WT. In addition, salt-induced K^+^ fluxes are mediated by PM H^+^-ATPase (Shabala and Cuin, 2008). Several studies reported that salt-tolerant cultivars exposed to salt stress have higher H^+^-ATPase activity and more negative membrane potential, thus inhibiting NaCl-induced K^+^ efflux through depolarization-activated K^+^ outward-rectifying (GORK) channels in barley and poplar (Chen *et al.*, 2007; Sun *et al.*, 2009). Our results showed that K^+^ efflux in salt-treated transgenic roots was significantly increased by amiloride or sodium orthovanadate. However, K^+^ efflux induced by NaCl shock was markedly lower in transgenic roots than in those of the WT. Moreover, the transgenic roots had lower electrolyte leakage (Supplementary Fig. S4A at *JXB* online) and higher osmotic potential compared with WT roots under salt stress. These findings suggested that *AtLOS5* overexpression could maintain membrane stability and regulate PM H^+^-ATPase activity and GORK channels to decrease K^+^ efflux under salt stress. Furthermore, the evident K^+^ efflux caused by NaCl in transgenic and WT roots was significantly reduced by the K^+^ channel blocker TEA (Supplementary Fig. S5). These results also indicated that salt-induced K^+^ efflux is mediated by the GORK channels or non-selective cation channels (NSCCs) (Shabala *et al.*, 2005; Shabala and Cuin, 2008).

Exposure of plants to salinity also leads to the oxidative damage initiated by reactive oxygen species (ROS), and ROS contribute to massive K^+^ leakage from the cell (Shabala and Pottosin, 2014). Several reports demonstrated that ROS can activate NSCCs (Demidchik *et al.*, 2003; Zepeda-Jazo *et al.*, 2011) and GORK channels (Demidchik, 2014). Proline has been widely proved to play an important role in salt tolerance by protecting protein and membrane structure (Verslues *et al.*, 2006). Exogenous proline can significantly reduce NaCl- and ROS-induced K^+^ efflux from barley roots, and maintain cytosolic K^+^ homeostasis through enhancing H^+^-ATPase activity and controlling GORK channels (Cuin and Shabala, 2005, 2007). Transgenic *AtLOS5* plants showed higher proline accumulation (Supplementary Fig. S4B at *JXB* online) under salt stress which might be involved in regulating K^+^ efflux by influencing H^+^-ATPase activity and GORK channels. High proline levels in transgenic plants could be the result of a protective effect on PM integrity and its associated transporter proteins under salt stress.

Salt stress inhibits plant water transport, and the *L*pr reduction caused by salt stress is an important plant response to minimize water stress (Boursiac *et al.*, 2005; Horie *et al.*, 2011). This process is accompanied by the regulation of the mRNA and protein level of most aquaporins, and aquaporin protein has been confirmed to be crucial for plants to combat salt stress (Boursiac *et al.*, 2005; Qian *et al.*, 2015). Although NaCl inhibits aquaporin-mediated root water transport, the regulation of *L*pr under salt stress is directly correlated with PIP proteins (Horie *et al.*, 2011). Salt stress significantly decreased root water potential and osmotic potential and reduced *L*pr in transgenic and WT plants (Fig. 10). Moreover, the up-regulation of *ZmPIP* transcripts at 6h after salt stress could indicate an effective regulatory mechanism to restore root water uptake (Zhu *et al.*, 2005; Qian *et al.*, 2015). Under prolonged salt stress, the transcripts of *ZmPIP* genes were down-regulated in transgenic and WT roots, as in several plant species (Boursiac *et al.*, 2005; Horie *et al.*, 2011). *AtLOS5* overexpression significantly increased root water potential, osmotic potential, and *L*pr under salt stress, together with the expression levels of *ZmPIP1:1*, *ZmPIP1:5*, and *ZmPIP2:4*. Since *L*pr is enhanced by application of exogenous ABA in maize (Ruiz-Lozano *et al.*, 2009; Marulanda *et al.*, 2010), the regulation of *L*pr by ABA might be linked to modulation of aquaporins, and ABA enhanced the expression of *ZmPIP* genes under stresses (Zhu *et al.*, 2005; Ruiz-Lozano *et al.*, 2009).

Plant responses to salinity include the osmotic and ionic phases of stress, and osmotic stress as a first component of salt stress (Munns, 2002; Munns and Tester, 2008). Detrimental effects of salinity are usually attributed to reduced water availability due to osmotic effects caused by the presence of high salt concentrations, and the reduction in leaf growth under salt stress is caused by a decrease in cell turgor as a result of osmotic stress (Munns and Tester, 2008). Leaf water potential and relative growth rate decreased as salt concentration increased, and salt stress progressively decreased leaf water potential and relative growth rate over time in both transgenic and WT plants, thus the relative growth rate was positively associated with leaf water potential (Supplementary Fig. S3 at *JXB* online) as reported previously (Bolaňos and Longsterth, 1984; Singh *et al.*, 2010). ABA has a dual function as a regulator of whole-plant water balance through transpiration and as a regulator of tissue water transport through water channel activity involved in growth responses to disturbances in external water potential (Hose *et al.*, 2000; Fricke *et al.*, 2006). Moreover, the significant increases in the concentrations of ABA in the leaves and roots under salt stress were in agreement with previous reports (Jia *et al.*, 2002; Fricke *et al.*, 2004; Costa *et al.*, 2007). While tissue ABA concentrations and water potentials were negatively correlated under salt stress, both were higher in transgenic plants than in the WT. Higher leaf water potential enhanced the relative growth rate and biomass accumulation in transgenic plants, in agreement with previous results (Fricke *et al.*, 2004; Costa *et al.*, 2007; Tardieu *et al.*, 2010), showing that the growth-promoting function of ABA was attributed to higher leaf water potential and increased hydraulic conductance.

Decreased hydraulic conductance is one of the first responses of plants to salt stress for reducing water loss (Boursiac *et al.*, 2005; Horie *et al.*, 2011). In this study, iso-osmotic PEG evoked a nearly identical reduction in *L*pr to that produced by 100mM NaCl. Moreover, *AtLOS5* overexpression significantly increased *L*pr under 100mM NaCl or iso-osmotic PEG. These findings indicated that transgenic *AtLOS5* plants had higher *L*pr which led to better water uptake for plants, leading to slight leaf wilting under salt stress. Moreover, transgenic plants showed smaller stomatal apertures and lower stomatal conductance than WT plants under salt stress (data not shown), which might maintain relatively higher water potential to reduce leaf wilting. Similar results were observed in transgenic *AtLOS5* maize plants exposed to drought stress (Lu *et al.*, 2013). In addition, iso-osmotic PEG caused ion fluxes that were similar to salt stress responses, which implied that salt-induced ion fluxes in maize were partly attributable to osmotic stress. Tolerance to osmotic stress is an important component of plant reaction to salt stress (Shavrukov, 2013). Accordingly, our results suggested that *AtLOS5* overexpression could enhance osmotic stress tolerance through regulating ion fluxes and water uptake under salt stress.

In summary, our data showed that *AtLOS5* overexpression could activate ABA biosynthetic genes and regulate AO activity to promote ABA accumulation in maize under salt stress, which could induce the expression and activity of ion transporters to regulate intracellular K^+^, H^+^, and Na^+^ fluxes for maintenance of a high cytosolic K^+^/Na^+^ ratio under salt stress. Concurrently, *AtLOS5* overexpression could modulate the *ZmPIP* gene transcripts involved in regulating the *L*pr to maintain better water uptake, which led to better water status for plant growth under salt stress. These results suggested that *AtLOS5* overexpression could enhance ABA biosynthesis to regulate the expression of ion transporters and PIP aquaporin to maintain a high cytosolic K^+^/Na^+^ ratio and better water status in maize under salt stress.

## Supplementary data

Supplementary data are available at *JXB* online.

Figure S1. RT-PCR analysis using *AtLOS5*-specific primers to identify the positive independent transgenic lines.

Figure S2. Effects of relative growth rate of transgenic and WT plants exposed to 0, 25, 50, 75, and 100mM NaCl treatments for 7 d. 

Figure S3. Effects of leaf water potential of transgenic and WT plants exposed to 0, 25 (A), 50 (B), 75 (C), and 100mM NaCl (D) treatments for 7 d. (E) The relationship between relative growth rate and leaf water potential.

Figure S4. The changes in electrolyte leakage and proline concentrations in transgenic and WT roots exposed to salt stress for 7 d. 

Figure S5. Effects of TEA on the net K^+^ fluxes in roots of transgenic and WT plants exposed to 100mM NaCl treatment for 2 d. 

Table S1. Primers used for qRT-PCR verification.

Supplementary Data
