# Supplementary material for: Increased abscisic acid levels in transgenic maize overexpressing AtLOS5 mediated root ion fluxes and leaf water status under salt stress
Source: J Exp Bot. 2016 Jan 7;67(5):1339–55. doi: 10.1093/jxb/erv528 (PMC4762378; doi:10.1093/jxb/erv528)
Supplement: Supplementary Data [file supp_67_5_1339__index.html]

Increased abscisic acid levels in transgenic maize overexpressing AtLOS5 mediated root ion fluxes and leaf water status under salt stress — Supplementary Data 

# Increased abscisic acid levels in transgenic maize overexpressing *AtLOS5* mediated root ion fluxes and leaf water status under salt stress

## Supplementary Data

Data files

- Supplementary\_Figure\_S1.tif - Supplementary Data
- Supplementary\_Figure\_S2.tif - Supplementary Data
- Supplementary\_Figure\_S3.tif - Supplementary Data
- Supplementary\_Figure\_S4.tif - Supplementary Data
- Supplementary\_Figure\_S5.tif - Supplementary Data
- Supplementary\_Table\_S1.pdf - Supplementary Data
