# Supplementary figures and images for: Increased abscisic acid levels in transgenic maize overexpressing AtLOS5 mediated root ion fluxes and leaf water status under salt stress
Source: J Exp Bot. 2016 Jan 7;67(5):1339–55. doi: 10.1093/jxb/erv528 (PMC4762378; doi:10.1093/jxb/erv528)

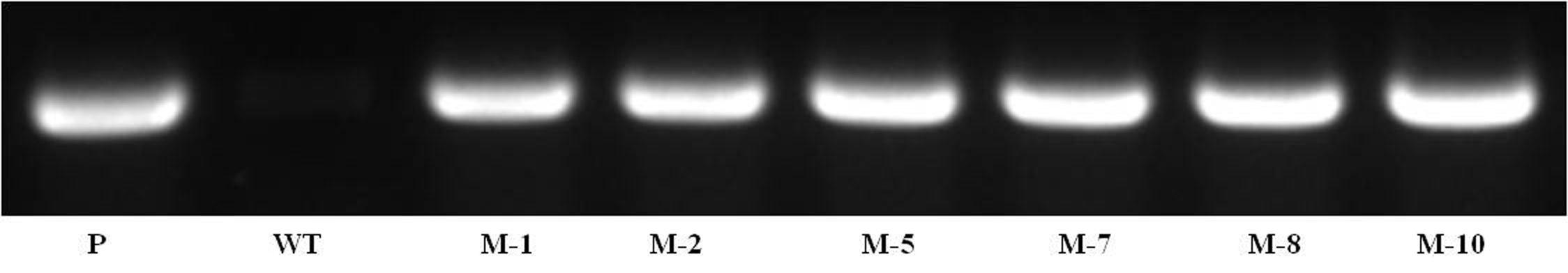

Supplement: Supplementary Data [file supp_erv528_Supplementary_Figure_S1.tif]

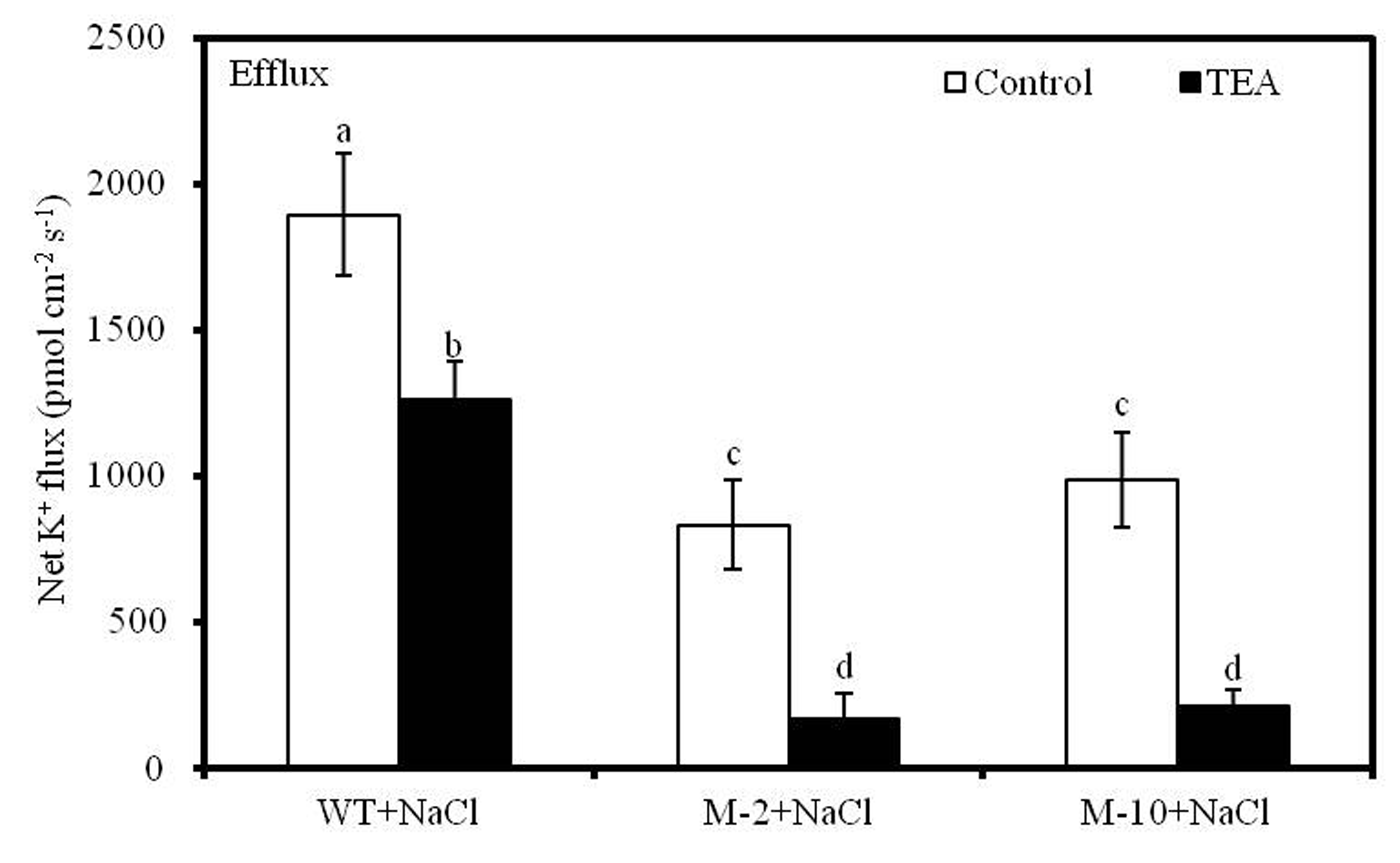

Supplement: Supplementary Data [file supp_erv528_Supplementary_Figure_S5.tif]
