# Supplementary material for: Increased abscisic acid levels in transgenic maize overexpressing AtLOS5 mediated root ion fluxes and leaf water status under salt stress
Source: J Exp Bot. 2016 Jan 7;67(5):1339–55. doi: 10.1093/jxb/erv528 (PMC4762378; doi:10.1093/jxb/erv528)
Supplement: Supplementary Data [file supp_erv528_Supplementary_Table_S1.pdf]

Table S1 Primers used for RT-qPCR verification.

Names	Forward primers	Reverse primers
AtLOS5	TGATGCTGCAAAGGGTT GTGCTAC	AATTGAAGCAGCAACAGTGCCTCC
ZmAO	GGGAGGCTGTGTACGTTGAT	TCTCCACCGCTTGGAATATC
ZmMOCO	CGGCAGGTGTACTTTGGGCAAA	CGGGGTCCTGATTCGGTCACTCAG
ZmVp14-2	GCCATCACCGAGAACTTGGT	CAGCATCTCCTGGAGCTTGAA
ZmNHX1	ATGCAGGGTTCCAAGTGAAG	AATATTGCCCCAAGTGCAAG
ZmCLB4	TCAGTGTGTTCCACCCTAAAGCA	ATCAAGCAGCGCCAAGACCAT
ZmCIPK16	CGATGACGCCCAAGAAAC	CCAGAGTTGCTGCTGCTGT
ZmPIP1-1	AGGTCTTAAAGGAGCCGATG	TGAACTCTTAAAGCTTGACTCG
ZmPIP1-5	GAGCCGTGACTGATTATACG	GGGAGTCTTCTTCTTAACTTTCA
ZmPIP2-4	GATCCGTCTGTGTTGATTTC	CGAACTGTGCATTGCATTTAT
β-actin	GATTCCTGGGATTGCCGAT	TCTGCTGCTGAAAAGTGCTGAG
